# Functional Food Consumption and Its Associated Factors among University Students in Malaysia during COVID-19 Pandemic

**DOI:** 10.21315/mjms2023.30.6.13

**Published:** 2023-12-19

**Authors:** Janice Ee Fang Tay, Serene En Hui Tung, Kai Ting Mok, Choon Hui Tan, Wan Ying Gan, Wai Chuen Poon

**Affiliations:** 1 Department of Food Science and Nutrition, UCSI University, Kuala Lumpur, Malaysia; 2 Division of Nutrition and Dietetics, School of Health Sciences, International Medical University, Kuala Lumpur, Malaysia; 3 Department of Nutrition, Faculty of Medicine and Health Sciences, Universiti Putra Malaysia, Selangor, Malaysia; 4 Sunway Business School, Sunway University, Subang Jaya, Malaysia

**Keywords:** functional food, nutrition knowledge, attitude, university student, COVID-19

## Abstract

**Background:**

In the current situation of COVID-19, dietary intake that incorporates functional foods may potentially be a preventive measure for defence against viral infection. This study aimed to determine the consumption of functional foods and its associated factors among university students during COVID-19.

**Methods:**

This was a cross-sectional study conducted among 284 Malaysian university students in Kuala Lumpur, Malaysia. An online self-administered questionnaire was employed to assess subjects’ nutrition knowledge, dietary habits, attitude towards functional foods, recognition and consumption of functional food products.

**Results:**

Out of 284 respondents, 41.9% had poor level of nutrition knowledge and 57% had moderate level of functional food-related attitude, with seven types of functional foods consumed on average (57.0%). Binary logistic regression showed that university students who consumed fruits at least three times per day (aOR = 11.18; 95% CI: 1.46, 80.17), salty snacks (aOR = 2.90; 95% CI: 1.43, 5.86), soft drinks/sugar-sweetened beverages (SSB) (aOR = 3.12; 95% CI: 1.53, 5.26) and pure juice (aOR = 2.80; 95% CI: 1.48, 5.30) were more likely to consume functional foods during COVID-19 (*P* < 0.05).

**Conclusion:**

The findings could provide information to public and private sectors in terms of creating a supportive environment to encourage and promote the awareness and consumption of functional foods and their associated health benefits.

## Introduction

Over the past few decades, food is no longer just to satisfy hunger; extra benefits such as enhancing one’s health status is also expected. As a result, there is an increased demand for food and beverage products with enhanced function that could improve or maintain health globally (1). In addition, the rising cost of healthcare, gradual increment in life expectancy, and the desire for a better quality of life, have become the main reasons for increased consumer awareness towards what they eat daily (1). As a result, functional food products have emerged in the market to respond to consumers’ demand for healthier options of food products.

Functional food was first introduced in Japan as ‘food for specified health use’ (FOSHU) in the late 1980s to show the relationship between nutrition, sensory satisfaction, fortification and modulation of the physiological system. Eventually, this concept entered the food market in the United States, Europe, Canada and China. Since then, the global market for functional foods has been growing at a fast pace with an annual average growth rate of approximately 8.5% and was estimated to be up to USD305.4 billion in the year 2020 (2). The emergence of functional food products in the market has catered consumers’ demand for healthier food product options that provide health benefits beyond basic nutrition such as bio-active compounds or fortified nutrients for physiological function improvement or reduction of disease risks (3).

With the current situation of COVID-19, the heightened fears around health and wellness are likely to prompt consumers to seek out alternate complementary remedies as it has been suggested that the host’s immune system could be the secret to defeating this virus (4). Recent studies suggested that non-communicable disease prevention through functional foods can be translated into protection against respiratory virus infections and COVID-19 (5). In particular, the immune-boosting effects found in functional foods and its bioactive components could help in controlling versatile physiological responses associated with virulent strain infections (6). For example, certain bioactive compounds within functional foods such as vitamins A, C and D, flavonoids and carotenoids, which are rich in antioxidants and anti-inflammatory agents, have been shown to enhance immune system functions and are considered to be advantageous in the prevention of COVID-19 (7). Likewise, both probiotics and prebiotics have been shown to be beneficial to the immune system by stimulating the growth and activity of gut microbiota (7). These show that functional foods may potentially be a good alternative complementary remedy for health management and protection during the pandemic (8).

Google Trends data has shown that online searches for the terms ‘food’ and ‘immune system’ have increased 670% globally in the first 2 weeks of March 2020, indicating a rising global interest in food products with immuneenhancing properties (9). Similarly, Mayasari et al. (10) demonstrated a moderate correlation between COVID-19 daily confirmed cases with immune-related nutrient and herb-related search terms using Google Trends. In addition, the impact of COVID-19 on mental health has led to the rise in demand for functional foods containing mood-boosting ingredients (9, 11).

Several factors have been acknowledged as agents influencing consumers’ consumption preference, acceptance and purchase intention towards functional foods including socioeconomic demography, perceived diet effectiveness of products, taste, lifestyle, cost, convenience and many more (12, 13). Consumers with positive attitude towards functional foods have higher willingness to try and accept functional foods (14). Sufficient nutrition knowledge is crucial in helping consumers to understand the potential beneficial effects of functional foods and to identify and distinguish the difference between conventional foods and functional foods (15). University students as young consumers are found to have higher interest and acceptance in consuming functional foods as they are more open-minded about novelty (14, 16). Therefore, it is certainly significant to identify the associated factors of functional food consumption as part of encouraging health-related behaviours among university students as potential consumers of functional foods during COVID-19 and in the future.

## Methods

### Study Design and Participants

This cross-sectional study was conducted among public and private university students aged 18 years old–30 years old in Klang Valley, Malaysia. The minimum required sample size for this study was determined using the rule-of-thumb formula for regression analysis as illustrated by Green (17),


(1) N≥104+m

A minimum sample size of 131 participants was required after consideration of a 10% nonresponse rate. Convenience sampling method was used through online platforms including academic social networking sites of universities and social media applications (Facebook and Instagram). Data collection was conducted between September 2020 and November 2020 during the Recovery Movement Control Order (RMCO) in Malaysia. International students and individuals with any physical and psychological disabilities or following a specific type of diet were excluded. Informed consent was obtained from all participants before answering the online questionnaire using Google Form. A total of 311 online questionnaires were collected; only 284 agreed and completed the questionnaires, producing a response rate of 91.31%.

### Measures

#### Socio-demographic Characteristics and Anthropometric Data

Socio-demographic characteristics including age, gender, ethnicity, academic qualification, current university and monthly allowance were collected. Anthropometric data including weight (kg) and height (cm) were self-reported by participants, which were further translated into Body Mass Index (BMI) and expressed as kg/m^2^ (18).

#### Recognition and Consumption of Functional Food Products

The recognisability and consumption of functional food categories were measured using the 10 functional foods listed in Salleh et al. (19). The selected product categories were bread, fruit juices, biscuits, milk, eggs, yogurts, cereal, margarine, soft drinks and sweets. Perceived familiarity was evaluated using a 5-point scale with response ranging from 1 = ‘I do not recognise this product’ to 5 = ‘I use this product frequently’ to assess participants’ level of familiarity and frequency of use of different product categories (20). The scale was further categorised into non-functional food users (1, 2 and 3) and functional food users (4 and 5). The number of types of functional foods consumed was determined based on the 10 categories

of functional foods. These were subsequently categorised into low (0–3 types), moderate (4–6 types) and high (7–10 types) in consumption. The Cronbach’s alpha coefficient for this section of the study was 0.807.

#### Nutrition Knowledge Level

Nutrition knowledge level was assessed using the questionnaire developed from past questionnaires (21, 23) and healthy eating elements with reference to the Malaysian Dietary Guidelines and the Malaysian Healthy Plate (24). The participants answered 30 multiple choice statements. One point was given for every correct response, while 0 was given to every incorrect or unsure response. The scores were further computed based on a maximum score of 30 and categorised into poor (≤ 59%), moderate (60%–79%), and good (≥ 80%) to rank the participants’ level of nutrition knowledge using Bloom’s cut-off points (25). The Cronbach’s alpha coefficient for this section of the study was 0.819.

#### Dietary Habits

Meal frequency was assessed using five items (breakfast, lunch, snacks, dinner and supper) adopted from the Eating Behaviour Questionnaire (26). This questionnaire was validated in a previous study (27). Participants were requested to indicate their meal frequency using a 6-point Likert scale response ranging from 1 = ‘Everyday’ to 6 = ‘Never at all’. The frequencies were further dichotomised into two categories: i) consumed everyday and ii) not consumed everyday.

Food group consumption was assessed using the questionnaire adopted from the Youth Risk Behaviour Survey (28). Participants were required to report the frequency of each food group consumed during the past 7 days using a 7-point scale with response ranging from 1 = ‘0 times during the past 7 days’ to 7 = ‘4 or more times per day’. Sweets, salty snacks, fast food, soda, sugar-sweetened beverages (SSB), 100% fruit juice and milk/dairy products were dichotomised into no consumption versus any consumption. Fruits and vegetables intake were dichotomised into three or more times per day versus less than three times per day. Chicken, meat, fish, beans and legumes, and bread, rice, noodles and rice flour categories were dichotomised into more than three times per week versus three or less than times per week (29). The Cronbach’s alpha coefficient for this section of the study was 0.786.

#### Functional Food-Related Attitudes

Functional food-related attitudes were assessed using a questionnaire adapted from Urala and Lähteenmäki (20). The original English version questionnaire was used. The definition for functional foods as suggested by Doyon and Labrecque (30) was included to help participants better understand what functional foods were. Participants were required to express their levels of agreement towards 26 statements describing four dimensions: i) reward (eight items), ii) necessity (nine items), iii) confidence (four items), and iv) safety (five items) through a 7-Likert scale with response ranging from 1 = ‘completely disagree’ to 7 = ‘completely agree’. It is to be noted that 12 items had a negative approach and to consolidate these questions, they were reversely scored. Based on the maximum score of 182, participants were categorised into low (≤ 59%), moderate (60%–80%) and high (≥ 81%). The Cronbach’s alpha coefficient for this section of the study was 0.905.

### Statistical Analysis

Data analysis was performed using the IBM SPSS version 24.0. Descriptive statistical analysis, including mean score, standard deviation, percentage and frequency were generated to summarise the distribution of the data. Chi-square statistical test of independence and Fisher’s exact test were used to estimate the association between categorial variables. Multiple logistic regression was performed to identify the predictors of functional food consumption among university students during the pandemic period, using socio-demographic variables (age, gender, ethnicity, academic qualification and monthly allowance) and BMI classification in the adjusted model. Statistical significance level for all analyses was set at *P <* 0.05.

## Results

### General Characteristics

Out of 284 participants, 74.6% were females, with a mean age of 22.5 ± 2.5 years old (Table 1). Majority of the participants were Chinese (82.4%) and pursuing a bachelor’s degree in education in the present study (69.7%), with a monthly allowance between RM1,000 and RM2,499 (52.8%). As for weight status, majority were normal in BMI (53.5%), followed by underweight (20.4%), overweight (17.3%) and obese (8.8%).

### Recognition and Consumption of Functional Food Products

**Figure 1:**
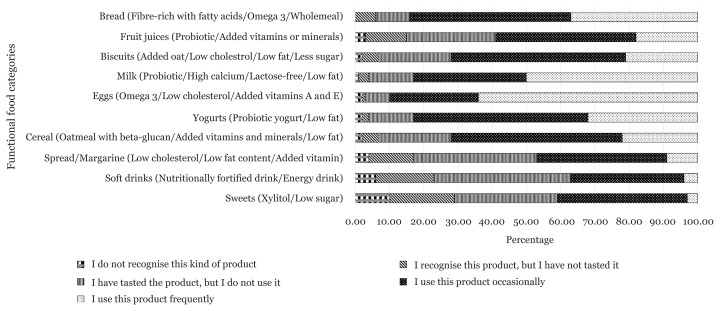
The recognition and consumption of functional food products during the pandemic

The recognition and consumption of functional food products during the pandemic is summarised in Figure 1. From the given list of 10 types of functional food products available in Malaysia, eggs enriched with nutrients such as Omega 3, vitamins A and D were the most consumed food products (64.4%), followed by milk products (50.0%). Less than a quarter of participants with the range of 1% to 19% stated that they recognised the listed products, but never tried before. Besides, xylitol or low-sugar sweets were the least recognisable and consumed functional foods among the participants (10.2%). Majority of participants reported that they consumed at least seven types of functional foods (57.0%), with an average of 6.70 ± 2.66 types of functional foods.

### Nutrition Knowledge Level and Functional Food-Related Attitudes

Most of the participants (41.9%) had poor levels of nutrition knowledge, 34.9% with moderate levels of nutrition knowledge and 23.2% with good levels of nutrition knowledge. As for functional food-related attitudes, most of the participants were found to have moderate-level attitude (57.0%), followed by low- (39.4%) and high-level (3.5%) attitudes (Table 2).

### Dietary Habits

More than half of the participants (61.6%) had meal skipping behaviours, especially skipping breakfast (49.6%). Besides, very few participants reported consuming fruits (4.9%) and vegetables (14.8%) at least three times per day for the past week. More than three-quarter of the participants consumed chicken and meats (85.9%), and also bread, rice, noodles and rice flour (86.3%) for three times or more in a week. Besides, less than half of the participants reported they had consumed fish (37.3%), beans and legumes (40.5%) at least three times for the past week. Participants were reported to consume sweets (83.1%), salty snacks (65.6%), soft drinks/SSB (53.5%), milk/dairy products (85.2%) over the past 7 days (Table 2).

### Factors Associated with Functional Food Usage

Table 3 demonstrates the factors associated with functional food usage during COVID-19 pandemic. There was a significant association between nutrition knowledge and the use of functional foods (χ^2^ = 14.33, *P* < 0.001), in which functional food users tend to have moderate levels of nutrition knowledge (43.7%). Interestingly, non-functional food users were those with high nutrition knowledge levels as compared to functional food users, which consisted of 31.5%. No significant association was found between functional food-related attitude and functional food users (*P* > 0.05).

As for dietary habits, the meal pattern and snack consumption between functional food users and non-functional food users were similar, except for supper, in which more functional food users (7.9%) reported that they consumed supper every day compared to non-functional food users (0.0%) (χ^2^ = 12.99, *P* < 0.001).

As presented in Table 3, a significant difference in proportion was found in the consumption of fruits, vegetables, salty snacks, fast foods, soft drinks/SSB, milk/dairy products and pure juice between functional food users and non-functional food users. More functional food users (9.5%) were reported to consume fruits and vegetables at least three times per day (χ^2^=10.200, *P* = 0.001) compared to nonfunctional food users (1.3%). In addition, significantly more functional food users consumed salty snacks (χ^2^ = 8.317, *P* = 0.004), fast food (χ^2^ = 4.068, *P* = 0.044) and soft drinks/SSB (χ^2^ = 10.550, *P* = 0.001) in the past week as compared to non-users. More functional food users reportedly had milk/dairy products (91.3%) (χ^2^ = 6.597, *P* = 0.010) and pure juice (57.9%) (χ^2^ = 28.599, *P* < 0.001) consumption in the past week compared to non-users.

### Predictors of the Use of Functional Foods

A binary logistic regression analysis was conducted to ascertain the extent to which the variables significantly predicted the likelihood of the use of functional foods (Table 3). General characteristics of participants were identified as covariates for binary logistic regression with functional food consumption (*P* < 0.25) (31): participants’ ethnicity and monthly allowance. In the present study, those consuming fruits at least three times per day were 11.2 times more likely to consume functional foods (aOR = 11.18; 95% CI = 1.46, 80.17). The likelihood of the use of functional foods was observed significantly among those who consumed salty snacks (aOR = 2.90; 95% CI = 1.43, 5.86), soft drinks/SSB (aOR = 3.12; 95% CI = 1.53, 5.26) and pure juice (aOR = 2.80; 95% CI = 1.48, 5.30) than those with no consumption of these products.

## Discussion

The findings in this study indicated that the participants presented a high recognition and consumption of functional food products during the COVID-19 pandemic, given that up to seven types of functional foods were consumed on a regular basis. This could be due to the pandemic outbreak that had raised overall health awareness and consciousness. This was supported by a past study (32), where a sharp rise of health consciousness was observed during the pandemic. Consumers tend to purchase food products perceived as healthier during strict lockdown period (33). During the pandemic, the public reported that they started to show stronger interest in nutrition by focusing on the nutritional characteristics and functional capacity of foods during their purchase. By improving healthy lifestyle behaviours, such as adopting a healthier diet, this may contribute to prevention of viral infections (34). When comparing with similar study during non-pandemic situation, the findings were parallel with a Korean study where 57.8% of college students consumed health functional foods (35). However, in other studies conducted in Italy, the United States and Turkey, there were inconsistent findings where poor recognisability of functional foods were observed (36, 37). This may be due to the fact that functional foods were not well-distinguished from conventional foods as similar distribution and marketing strategies were used (38).

University life is well recognised as one of the critical periods for young adults as they are more susceptible to experience changes, including adopting new nutritional behaviours. University students are constantly pressured to make food choice decisions that are considered to be healthier during their transition from secondary school to higher education as independence increases. Generally, university students’ diets are not nutritionally balanced or sufficient, and are often characterised as irregular meals, snacking, skipping breakfast and reduced fruits and vegetables consumption (39). It is possible that formation of such trends due to irregular class timetables may also influence the dietary habits among university students, particularly skipping meals when class times are scheduled during breakfast or lunch hours (40). It should be highlighted that poor dietary habits formulated during university period have considerable implications on health of individuals throughout life. Therefore, it is essential to understand the importance of healthy dietary habits that should be consistently adopted.

COVID-19 pandemic further worsened the problem of poor dietary habits by putting more pressure on people to seek out and follow a nutritious diet. More than two-third of participants in the present study did not consume three main meals daily on a regular basis, given that their fruits and vegetables consumption did not adhere to the Malaysian

Dietary Guidelines 2020 (24). Findings from a past study concluded that the diet intake and quality among university students during the COVID-19 pandemic period was observed to be poorer than before pandemic due to decreased consumption of nutrient-dense foods including grains, fruits, vegetables, dairy, nuts, meat and meat alternatives, which reflected a decrease in total caloric intake (41). In contrast, an Australian study by Gallo et al. (42) observed a significant increase in calorie intake by about 20% among university students during the confinement measures as a result of increased frequency of snacking.

Despite the inconsistent findings across the studies, it can still be concluded that the COVID-19 pandemic confinement had a negative impact on food consumption and meal patterns. Nevertheless, Scarmozzino and Visioli (43) stated that majority of their participants (49.6%) among the Italian population showed no significant changes in diet during lockdown. A nutrient-dense diet is highly important in maintaining physiological health and to support the immune system to reduce the susceptibility towards diseases, especially during adverse events, including the COVID-19 pandemic. As such, confinement during COVID-19 that has resulted in the closures of businesses and reduced hours of operation of food stores can limit shopping frequency, at-home food availability, and the accessibility to community and organisational food environments (41); thus, leading to unfavourable food choices and dietary habits.

Logistic regression analysis performed in the study identified the significant factors associated with functional food consumption during COVID-19 pandemic. Some of the findings included consuming fruits at least three times per day, consuming salty snacks, soft drinks/SSB and pure juice for the past week during COVID-19 pandemic, with consideration of ethnicities and monthly allowance. Consumption of fruits and pure fruit juices were generally observed among individuals with high health awareness and consciousness. In fact, this group of individuals have a higher acceptability and are more inclined to consume healthy foods including functional foods (44). A past study by Mullie et al. (45) concluded that functional food consumption was associated with healthy dietary pattern, which is characterised as high consumption of tomatoes, fruits, low-fat dairy products, whole grains, vegetables, cold breakfast cereals, fruit juice, fish, tea and nuts. However, this relation could not be confirmed in the present study as both salty snacks and soft drinks/SSB consumption were also found to be associated with the use of functional foods; and these foods are high in sugar and sodium, which are widely proven for their negative effects on human health, particularly increased risk of non-communicable diseases (46).

Interestingly, findings in the present study indicated that nutrition knowledge had no substantial impact on the use of functional foods during the COVID-19 pandemic. Theoretically, nutrition knowledge plays a vital role in determining an individual’s consumption behaviour (38). Individuals with higher levels of health- and nutrition-related knowledge are therefore more likely to consume functional foods as they have been indirectly influenced to have a degree of trust and confidence in functional foods (13). It is to be noted that the level of knowledge concerning functional foods including attribute-related knowledge and consequence-related knowledge are more likely to be the key determinants for functional food consumption compared to general nutrition knowledge, which could be the reason of the null association found between knowledge and functional food consumption in the present study. In other words, consumers would only consume functional foods if they perceive that functional foods are beneficial to their health (47). Additionally, consumers may perceive functional foods as processed foods, as in the NOVA food classification (48), a system which only emphasises on the degree of processing and does not account for the nutritional values and health benefits of the food itself. Therefore, consumers may be misled and thus avoid consuming functional foods (49). According to a local study by Hassan et al. (50), Malaysians are still certain that nutritious foods must be fresh and perishable and have little knowledge of healthy processed foods that should be also regularly consumed. Therefore, there is a need to increase consumer understanding on the need of functional foods as basic processed food necessity.

This study is not without limitations. As the participants in the study were recruited using convenience sampling, the results may not be representative of the Malaysian university students as a whole. In addition, the data collected were self-reported, which were highly dependent on the participant’s memory, honesty and truthfulness in providing answers. Besides, the study did not completely capture the actual consumption of functional foods, but only rated their levels of familiarity and consumption. To further ascertain the accuracy of functional food consumption, it is recommended to assess the types, brands, frequency, and amount of consumption. Despite the questionnaires in the present study showing acceptable levels of reliability, further validation tests are suggested to ensure the credibility of the questionnaires.

## Conclusion

The main aim of the study was to identify the associated factors of functional food consumption among university students and significant findings were demonstrated. This study revealed the increasing trend of functional food consumption during the COVID-19 pandemic among university students, in which individuals prioritised personal health management through a sufficient and nutritious diet in order to effectively improve their immune system and fight diseases. Results demonstrated that dietary patterns such as fruits (≥ three times per day), salty snacks, soft drinks/SSB and pure juice consumption were predictors of the use of functional foods during the COVID-19 pandemic. The findings could have implications for public and private sectors in terms of creating a supportive environment to encourage and promote the awareness and consumption of functional foods and their associated health benefits, thereby achieving optimal health status.

## Figures and Tables

**Table 1:** General characteristics of participants (*N* = 284)

	Total	Functional food user		*P*-value^a^
	
	(*N* = 284)	Non-user (*n* = 158)	User (*n* = 126)	
	
	*n* (%)	*n* (%)	*n* (%)	
Age (mean ± SD) years old	22.6 ± 2.5	22.3 ± 1.2	22.9 ± 2.8	0.046*
Gender				0.772
Male	72 (25.4)	39 (24.7)	33 (26.2)	
Female	212 (74.6)	119 (75.3)	77 (73.8)	
Ethnicity				0.016*
Chinese	234 (82.4)	139 (88.0)	95 (75.4)	
Malay	25 (8.8)	8 (5.1)	17 (13.5)	
Indian	17 (6.0)	9 (5.7)	8 (6.3)	
Others	8 (2.8)	2 (1.3)	6 (4.8)	
Academic qualification				0.816
Foundation/A-level/Diploma or equivalent	61 (21.5)	33 (20.9)	28 (22.2)	
Bachelor’s degree	198 (69.7)	112 (70.9)	86 (68.3)	
Master’s degree	19 (6.7)	9 (5.7)	10 (7.9)	
PhD	6 (2.1)	4 (2.5)	2 (1.6)	
Monthly allowance (RM)				<0.001*
< 1,000	76 (26.8)	57 (36.1)	19 (15.1)	
1,000–2,499	150 (52.8)	75 (47.5)	75 (59.5)	
2,500–3,999	35 (12.3)	20 (12.7)	15 (11.9)	
≥4,000	23 (8.1)	6 (3.8)	17 (13.5)	
BMI classification				0.357
Underweight	58 (20.4)	29 (18.4)	29 (23.0)	
Normal weight	152 (53.5)	92 (58.2)	60 (47.6)	
Overweight	49 (17.3)	25 (15.8)	24 (19.0)	
Obese	25 (8.8)	12 (7.6)	13 (10.3)	

Notes: Data were expressed as *n* (%) unless otherwise indicated; Significant association was measured by ^a^chi-square statistical test; Significant difference was measured by Independent *T*-test;

* Significant at *P* < 0.05

**Table 2:** Associations between selected variables and functional food usage (*N* = 284)

Variables	Total (*N* = 284)	Functional food user		*X*^2^ statistics (df)	*P*-value

		Non-user (*n* = 158)	User (*n* = 126)		

	*n* (%)	*n* (%)	*n* (%)		
Nutrition knowledge level					
Poor	119 (41.9)	65 (41.1)	54 (42.9)	14.330 (2)	< 0.001^a^*
Moderate	99 (34.9)	44 (27.8)	55 (43.7)		
Good	66 (23.2)	49 (31.0)	17 (13.5)		
Functional food-related attitude					
Low	112 (39.4)	66 (41.8)	46 (36.5)	3.186 (2)	0.203^a^
Moderate	162 (57.0)	89 (56.3)	73 (57.9)		
High	10 (3.5)	3 (1.9)	7 (5.6)		
Dietary habits:					
Meal and snack consumption					
Breakfast everyday					
Yes	143 (50.4)	76 (48.1)	67 (53.2)	0.722 (1)	0.396^a^
No	141 (49.6)	82 (51.9)	59 (46.8)		
Lunch everyday					
Yes	247 (87.0)	140 (88.6)	107 (84.9)	0.841 (1)	0.359^a^
No	37 (13.0)	18 (11.4)	19 (15.1)		
Snacking everyday					
Yes	44 (15.5)	22 (13.9)	22 (17.5)	0.670 (1)	0.413^a^
No	240 (84.5)	136 (86.1)	104 (82.5)		
Dinner everyday					
Yes	234 (82.4)	128 (81.0)	106 (84.1)	0.469 (1)	0.494^a^
No	50 (17.6)	30 (19.0)	20 (15.9)		
Supper everyday					
Yes	10 (3.5)	0 (0.0)	10 (7.9)	12.997 (1)	< 0.001^b^*
No	274 (96.5)	158 (100.0)	116 (92.1)		
Meal skipping behaviour					
Yes	175 (61.6)	104 (65.8)	71 (56.3)	2.660 (1)	0.103^a^
No	109 (38.4)	54 (34.2)	55 (43.7)		
Frequency of food groups consumption in past week					
Fruits					
< three times/day	270 (95.1)	156 (98.7)	114 (90.5)	10.200(1)	0.001^a^*
≥ three times/day	14 (4.9)	2 (1.3)	12 (9.5)		
Vegetables					
< three times/day	242 (85.2)	141(89.2)	101 (80.2)	4.588 (1)	0.032^a^*
≥ three times/day	42 (14.8)	17 (10.8)	25 (19.8)		
Chicken and meats (beef, lamb or pork)					
≤ three times/week	40 (14.1)	23 (14.6)	17 (13.5)	0.066 (1)	0.798^a^
> three times/week	244 (85.9)	135 (85.4)	109 (86.5)		
Fish					
≤ three times/week	177 (62.3)	101 (63.9)	76 (60.3)	0.388 (1)	0.533^a^
> three times/week	107 (37.7)	57 (36.1)	50 (39.7)		
Beans and legumes					
≤ three times/week	169 (59.5)	101 (63.9)	68 (54.0)	2.883 (1)	0.089^a^
> three times/week	115 (40.5)	57 (36.1)	58 (46.0)		
Bread, rice, noodles and rice flour					
≤ three times/week	39 (13.7)	27 (17.1)	12 (9.5)	3.386 (1)	0.066^a^
> three times/week	245 (86.3)	131 (82.9)	114 (90.5)		
Any sweet consumption					
Yes	231 (81.3)	130 (82.3)	101 (80.2)	0.208 (1)	0.649^a^
No	53 (18.7)	28 (17.1)	25 (19.8)		
Any salty snacks consumption					
Yes	186 (65.5)	92 (58.2)	94 (74.6)	8.317 (1)	0.004^a^*
No	98(34.5)	66 (41.8)	32 (25.4)		
Any fast-food consumption					
Yes	141 (49.6).	70 (44.3)	71 (56.3)	4.068 (1)	0.044^a^*
No	143 (50.4)	88 (55.7)	55 (43.7)		
Any soft drinks/SSB consumption					
Yes	152 (53.5)	71 (44.9)	81 (64.3)	10.550 (1)	0.001^a^*
No	132 (46.5)	87 (55.1)	45 (35.7)		
Any milk/dairy products consumption					
Yes	242 (85.2)	127 (80.4)	115 (91.3)	6.597 (1)	0.010^a^*
No	42 (14.8)	31 (19.6)	11 (8.7)		
Any pure juice consumption					
Yes	115 (40.5)	42 (26.6)	73 (57.9)	28.599 (1)	< 0.001^a^*
No	169 (59.5)	116 (73.4)	53 (42.1)		

Notes: Data were expressed as *n* (%) unless otherwise indicated;

* Significant association was measured by

a chi-square statistical test and

b Fisher’s exact test

**Table 3:** Associated factors of functional food consumption among university students

Variables	Unadjusted model^a^			Adjusted model^a^		
	
	OR	95% CI	*P*-value	OR	95% CI	*P*-value
Nutrition knowledge						
Poor	Reference			Reference		
Moderate	1.83	(0.99, 2.37)	0.053	2.20	(1.06, 4.56)	0.054
Good	0.44	(0.21, 0.94)	0.034*	0.48	(0.19, 1.20)	0.118
Dietary habits						
Fruits consumption						
< three times/day	Reference			Reference		
≥ three times/day	10.15	(1.70, 60.66)	0.011*	11.18	(1.56, 80.17)	0.016*
Vegetables consumption						
< three times/day	Reference			Reference		
≥ three times/day	0.94	(0.38, 2.35)	0.899*	0.88	(0.32, 2.39)	0.794
Any salty snacks consumption						
No	Reference					
Yes	1.93	(1.05, 3.54)	0.034*	2.90*	(1.43, 5.86)	0.003*
Any fast-food consumption						
No	Reference					
Yes	0.88	(0.49, 1.58)	0.669*	0.79	(0.39, 1.61)	0.515
Any soft drinks/SSB consumption						
No	Reference			Reference		
Yes	2.06	(1.15, 3.69)	0.015*	3.12	(1.53, 5.26)	0.002*
Any milk/dairy products consumption						
No	Reference			Reference		
Yes	1.78	(0.78, 4.06)	0.173*	1.70	(0.63, 4.61)	0.295
Any pure juice consumption						
No	Reference			Reference		
Yes	2.98	(1.71, 5.20)	< 0.001*	2.80	(1.48, 5.30)	0.002*

Notes: Estimates of odds ratio from abinary logistic regression adjusted ethnicity and monthly allowance;

* Statistical significant at *P* < 0.05;

OR = odds ratio; CI = confidence interval
